# Hepatitis B in Healthcare Personnel: An Update on the Global Landscape

**DOI:** 10.3390/v15122454

**Published:** 2023-12-18

**Authors:** Georgia B. Nikolopoulou, Ioannis Tzoutzas, Athanasios Tsakris, Helena C. Maltezou

**Affiliations:** 1 Department of Hepatitis, National Public Health Organization, 3-5 Agrafon Street, 15123 Athens, Greece; g.nikolopoulou@eody.gov.gr; 2 School of Dentistry, National and Kapodistrian University of Athens, 2 Thivon Street, 11527 Athens, Greece; tzoudent@dent.uoa.gr; 3 Department of Microbiology, School of Medicine, National and Kapodistrian University of Athens, 75 Mikras Asias Street, 11527 Athens, Greece; atsakris@med.uoa.gr; 4 Directorate of Research, Studies and Documentation, National Public Health Organization, 3-5 Agrafon Street, 15123 Athens, Greece

**Keywords:** hepatitis B, hepatitis B virus, health care personnel, health care workers, vaccination, occupational risk, needle stick and sharp injuries

## Abstract

Despite the outstanding progress that has been made in the prevention, detection, and management of hepatitis B during the past decades, hepatitis B remains a problem among healthcare personnel (HCP) in many countries. We reviewed studies on all aspects of hepatitis B in HCP published from 2017 through April 2023. They revealed wide variations on the prevalence of infection among HCP, ranging from 0.6% in Europe to >8.7% in Africa, almost always in association with very low vaccination rates. Many studies found a significant association between HCP’s knowledge about hepatitis B and hepatitis B vaccines, their vaccination status, and practices. This research also discloses global inequities regarding vaccination policies against hepatitis B, free-of-charge vaccinations, and access to post-exposure prophylaxis (PEP). Strategies to prevent and manage accidental exposures are needed in order to reduce the burden of hepatitis B on HCP, while written policies for all aspects of infection prevention, protective equipment, and PEP should be available. Lastly, HCP should be accordingly educated. These are all imperative given the decline of routine vaccinations in the COVID-19 era, particularly in countries with fragile vaccination programs, and the disruptions of interventions for hepatitis B that are expected to provide a pool of virus transmission to future generations.

## 1. Introduction

Despite advances in hepatitis B prevention and treatment, hepatitis B remains a public health problem with high morbidity and mortality worldwide. According to the World Health Organization (WHO), approximately 296 million individuals globally were living with hepatitis B infection in 2019. Asia and sub-Saharan Africa bear most of the burden of chronic hepatitis B, with a large proportion of countries being endemic with an estimated HBV prevalence of >8% [1].

Healthcare personnel (HCP) are at increased risk of hepatitis B virus (HBV) infection because of their frequent exposure to blood and other body fluids [2,3,4]. Once infected, HCP may transmit HBV to their patients [5]; however, with appropriate precautions, transmission is rare [6]. Because of their increased occupational risk, the WHO recommends that HCP are vaccinated against HBV [7]. Specifically, the hepatitis B vaccine confers over 96% protection, making HBV infection a vaccine-preventable disease. Nevertheless, hepatitis B vaccination rates among HCP are suboptimal in several countries, with low completion rates of the recommended three-dose vaccine series, while HCP uncommonly check their hepatitis B antibody levels after vaccination [8,9,10,11,12,13].

In order to plan efficient strategies for the containment of hepatitis B and guide health authorities, knowledge of the prevalence of HBV infection among HCP is necessary. To our knowledge, there are no recent review articles on hepatitis B in HCP. The present study reviews the recent published evidence on the prevalence of HBV infection and vaccination status in HCP worldwide. Aspects of vaccination policies against hepatitis B and infection prevention strategies are also discussed.

## 2. Methods

The PubMed search engine was searched for articles published from 2017 through 30 April 2023 using the following combination of words: “hepatitis B AND health care personnel”. Original and review English-language articles were eligible, regardless of the dates of data collection. We read the abstracts of a total of 430 articles identified through this first search round and selected 101 articles presenting data on HCP. In addition, information from two official public health websites and seven articles on occupational infections and vaccinations of HCP were used. In total, 111 references were included in the review.

Hepatitis B infection is defined as a HBsAg-positive and/or HBV DNA-positive laboratory result. Past hepatitis B infection is defined as a HBsAg-negative and anti-core-positive laboratory result. Full hepatitis B vaccination is defined as a primary vaccination series of three vaccine doses.

### 2.1. Immunity Status against Hepatitis B of HCP

A total of 24 studies reporting original data on hepatitis B prevalence were found [2,3,4,8,9,10,11,12,13,14,15,16,17,18,19,20,21,22,23,24,25,26,27,28]; among them, sixteen studies were conducted in Africa, six in Asia, and two in Europe. In addition, data from four review articles were included in the analysis: one review of studies conducted in Europe [29]; one systematic review and meta-analysis of studies conducted in Africa and Asia [30]; one systematic review and meta-analysis of studies conducted in Africa [31]; and an updated systematic review and meta- analysis of studies conducted in Ethiopia [32]. The characteristics and main findings of these studies are shown in Table 1.

The prevalence of hepatitis B infection in HCP was found to be low in Europe (0.6–1.2%) [29] and in some Asian countries (e.g., India, Iran, Turkey, and United Arab Emirates) [12,14,21,27]. However, the prevalence was much higher in other countries of Asia (up to 9.85%) [15] and in Africa (2.3–11.8%) [13,19]. In Asia, the highest prevalence was found in Vietnam [15]. In particular, two studies conducted in Vietnam showed that dentists had lower HBsAg positivity rates than other groups of HCP; however, even the dentist population showed a prevalence of 5%, which is still considered high [15,20]. An alarmingly high HBsAg prevalence rate of >8.7% has been reported in HCP in Cameroon, Mauritania, Sierra Leone, and Tanzania [8,10,11,13,17,22].

The difference in prevalence rates among countries is mainly explained by differences in the routine HBV vaccination policies between them, which in turn may influence immunity. For instance, East Asian countries, Europe, and North America vaccinate at birth; in sub-Saharan Africa, HBV vaccination is usually conducted at six weeks of age; and in some countries, vaccination or boosting is done in adolescence. Nevertheless, despite the WHO vaccination recommendations and national immunization guidelines, there are gaps in HCP vaccinations against hepatitis B. Reported completion rates of all three HBV vaccine doses is very low in low-income African countries (e.g., 4.3% in Sierra Leone and 9.1% in Cameroon) [11,17]. In Kenya, the percentage of vaccination was much higher, but while 80% of HCP had been vaccinated with at least one vaccine dose, only 48% had received all three doses [9]. This may be due to the unaffordability and/or unavailability of the vaccine, due to forgetting the subsequent doses, or because institutions only provide a partial number of doses. In Iran, 100% of HCP have been fully vaccinated [21]. In India, HCP are fully vaccinated at a high percentage (79.5%), but housekeeping staff is vaccinated only at a rate of 6.9%, indicating that considerable efforts should be made to educate and protect certain HCP groups [12]. In Vietnam, even medical professionals report low levels of vaccination, as only 15% of dentists and dental HCP were vaccinated, and 66.8% were anti-HBs negative and therefore susceptible to HBV [20].

### 2.2. Sex

Differences in HBV prevalence rates based on sex has not always clearly surfaced in the literature. In most studies, no difference in prevalence was found between male and female HCP [4,11,13,15,16,24,28]. In other studies, an increased proportion of HBV was detected in males, but the difference was not statistically significant [20,26]. The difference reached significance in Cameroon where women exhibited 40% lower prevalence rates than men and in Nigeria where male HCP were almost twice more likely to test positive to HBV infection than female HCP [10,19]. Two studies showed that female HCP were less likely to be exposed to blood or body fluids or needle stick and sharp injuries (NSSIs) compared to male HCP [3,20] and two others showed that female HCP had higher vaccination rates [14,27]. Only one study showed that HBV prevalence was higher among females [17].

### 2.3. Type of HCP Profession and Education

Two important factors affecting HBV prevalence are occupation (medical versus non-medical profession) and education. In general, knowledge and awareness of hepatitis B infection, the associated risks, and the measures to prevent it, have been associated with a lower HBV prevalence [19,30]. This knowledge has been reported to be higher and more systematic among medical personnel, compared with non-medical personnel [12,19,22]. Perhaps related to the level of knowledge, among other factors, the prevalence of hepatitis B infection among medical personnel in Tanzania was 9.1%, while among non-medical personnel, the prevalence was 30% [13]. In Cameroon, patient transporters were found to have a much higher HBV prevalence (14.3%) than physicians (3.2%) [10]. Nevertheless, there are exceptions to that pattern. In a study conducted in Northern Tanzania, the majority of HBsAg-positive HCP were physicians (24%), followed by nurses (20%), and laboratory personnel (16%) [24]. In Mozambique, laboratory technicians had the highest HBV prevalence [2]. A study conducted in a tertiary teaching hospital and a military general hospital in Sierra Leone found no significant difference in prevalence by sex, age, site of work, or type of profession [25].

In addition, NSSIs are a high-risk factor for HBV transmission. Therefore, HCP involved in intervention work-type (such as HCP with exposure-prone procedures) are at high risk [2,4,18]. However, in a seroprevalence study that was conducted in public hospitals in Iran, although NSSIs were reported by 42.4% of HCP, the HBV prevalence was very low (0.78%) which was attributed to their 100% vaccination rate [21].

Lastly, two studies found a correlation between the duration of employment and HBV seropositivity. In particular, HCP who had longer work experience were more likely to be seropositive to HBV than those that have worked for less time [19,23].

### 2.4. Knowledge and Attitudes of HCP towards Hepatitis B and Hepatitis B Vaccine

Recent studies indicate that gaps in knowledge about hepatitis B and the transmission of infection, lack of awareness of their HBV serostatus, and concerns about the safety and efficacy of hepatitis B vaccines, are still frequent among HCP [3,11,13,15,19,22,33,34,35,36,37,38,39,40,41,42,43,44,45,46,47]. For instance, a study among 300 HCP in India found that only 35.3% of them were aware of HBV transmission by NSSIs, 40% identified appropriate precautions, 17% identified the correct steps to disinfect a blood splash, and 54.7% were aware of post-exposure prophylaxis (PEP) [40]. Despite high rates of NSSIs, only half of nurses, midwives, and other HCP groups in hospitals in Sudan and the Democratic Republic of Congo were aware of PEP and the prevention of blood-borne infections [44,47]. Similarly, a survey conducted among 1,044 hospital-based HCP, including trainees, in 12 African countries (Cameroon, Egypt, Ethiopia, Ghana, Kenya, Madagascar, Malawi, Nigeria, Sierra Leone, Sudan, Tanzania, and Uganda) revealed gaps in their knowledge about HBV infection and low awareness of their HBV serostatus; nevertheless, 95% of participants (99% of medical trainees and nurses) were aware of the increased occupational risk for HBV infection [48]. Overall, significant differences in HCP’s knowledge about HBV infection and prevention and attitudes towards hepatitis B vaccines are recorded by geographic region, profession, and healthcare setting [11,37,39,47,48,49,50].

Studies from many countries, including high-HBV prevalence countries, indicate that HCP’s knowledge and perceptions about hepatitis B and hepatitis B vaccination determines their vaccination status, and overall attitudes and practices towards HBV infection, along with their demographic and professional characteristics and workplace experiences (e.g., age, sex, profession, years of education, years of employment, healthcare setting, and NSSIs) [22,34,41,44,46,47,51,52,53,54,55,56,57,58,59,60,61,62,63,64,65,66]. For instance, 31% of 81 HCP who refused HBV vaccination in a healthcare facility in Egypt did so because of a fear of vaccine side effects, while doubts about vaccine efficacy were also reported by 22.2% of them [50]. A 2019 survey in a tertiary care hospital in Ghana found that HCP with low knowledge about HBV infectivity were more likely to be unvaccinated by an adjusted odds ratio (OR) of 8.63 [95% confidence intervals (CI): 2.99–24.94] [56]. In contrast, a cross-sectional survey among 423 HCP in South Ethiopia showed that female HCP, those with more than ten years of work experience, and HCP in governmental healthcare facilities were more likely to be fully vaccinated (adjusted OR: 3.84, 12.51, and 2.45, respectively) [65]. Similarly, a survey among HCP employed in healthcare facilities in Uganda found a clear association between a belief that the hepatitis B vaccine is safe and being fully vaccinated against hepatitis B [57].

A large review of studies from Asia and Africa published from 1997 through September 2021 investigated the association between HCP’s HBV seroprevalence and level of knowledge or awareness of HBV infection [30]. Eleven of these studies found an association between HBV seroprevalence and level of awareness (good in nine studies and poor in two studies) [30]. Nevertheless, three studies found high HBV seroprevalence rates despite strong knowledge and awareness of HBV infection [30]. In this review, the overall HBV seroprevalence among 10,043 tested HCP was 5% in Africa and 4% in Asia [30]. 

Studies among dentists and other dental HCP also reveal insufficient knowledge about risk factors for the transmission of blood-borne infections, universal safety precautions, and PEP; these studies also show significant correlations between their knowledge about HBV, year of graduation, years of work experience, and workplace, which subsequently impact their attitudes and practices [36,37,61,67,68]. For instance, a cross-sectional study conducted among dental students in a teaching hospital in Nepal in 2019 found that only 10% of them were aware of their HBV serostatus, while most students lacked knowledge about the transmission of HBV infection, prevention, and PEP (e.g., only 11% and 13% of responders were aware of the importance of instrument sterilization and appropriate instrument disposal, respectively) [69]. In a study in four teaching hospitals in Pakistan where HBV-positive patients routinely receive dental care, more than 50% of dentists did not practice standard precautions for every patient [36]. Furthermore, a study among dental HCP in Georgia found that only 34.2% of participants correctly responded about risk factors for HBV transmission and only 37.3% reported being well-informed on infection control guidelines [68]. However, almost all (95.6%) dental HCP were interested in receiving additional education on blood-borne infections [68]. It is noteworthy that although 84.4% (1540 of 1824) of medical students in clinical training in Mexico had received some training on safe healthcare delivery and the use of PPE, only one out of three who had a NSSI received medical advice at the point of care [43].

In countries with high HBV prevalence, gaps in every-day practice in association with low vaccine uptake can prove detrimental. For instance, 68.8% of 358 nursing students recapped needles after injection in a survey in Ghana [70]. Similarly, up to 48.2% of 314 HCP in healthcare facilities in Vietnam consistently recap needles, while 24.2% of them reported a NSSI in the past 12 months [39]. Likewise, a retrospective study in 2016–2018 found that NSSIs were common among HCP in a medical complex in Saudi Arabia, with an overall incidence rate of 8.4% over 26 months [71]. These findings indicate discrepancies in the curriculum of medical, dental, and other healthcare schools in many countries; these findings also underline the need for post-graduate educational activities to increase the infection prevention capacity and build confidence about the benefits and safety of hepatitis B vaccines [15,39,46,47,49,57,63,72,73].

### 2.5. Vaccination Policies against Hepatitis B

Almost all high-income, developed countries have national vaccination policies against hepatitis B for HCP [74,75]. However, a recent survey conducted by the WHO found that only 55 of 103 (53.4%) responding WHO Member States (of a total of 194 invited WHO Member States) had or plan to have a national policy for vaccinating HCP against hepatitis B in the next five years [76]. In this study, there was a clear association between the existence of a vaccination policy for HCP and the income of the country [76]. However, efforts to improve full HBV vaccination rates at birth or early infancy is the best means to ensure future HCP are protected.

Even when national vaccination policies are in place, many studies in Africa and Asia indicate that vaccine availability and costs are key barriers for HCP vaccination against hepatitis B [41,48,51,54,55,56,62,64,65,77,78,79]. Although willingness to pay for their own vaccination was reported by 62.4% of HCP in governmental healthcare facilities in Ethiopia, the cost that they could afford was significantly less than the market price [78]. In contrast, a study among physicians and nurses in 22 urban hospitals in China found significantly higher vaccination rates against hepatitis B in three hospitals with a free vaccination policy (91.7% versus 79.0%; *p*-value < 0.001) [63]. Free-of-charge and easy access to vaccinations are key points to raise vaccine uptake among HCP.

Not having been informed about vaccination, forgetting the dates of vaccinations, and a complicated vaccination process have also been identified as reasons for not receiving vaccine doses [48,79,80,81]. Overall, vaccine hesitancy among HCP should be regarded in the context of intervention hesitancy, which addresses hesitancy towards a spectrum of infection prevention interventions [82]. Intervention hesitancy is impacted by poor employment conditions and mistrust in health authorities along with staff and resource shortages [82].

The most critical time to promote HBV screening and the vaccination of HCP is upon employment. Such a policy will need the allocation of financial and human resources but also support by employers and governments [83]. A policy requiring vaccination against hepatitis B for job entry has been the major driver for full vaccination among 252 HCP in two Pakistani hospitals with an adjusted odds ratio (OR) of 4.6 [95% confidence interval (CI): 1.5–5.3] [34]. The vaccination of preclinical students can also ensure their protection throughout their profession life [72].

Moreover, health authorities should explore the feasibility of mandatory vaccination policies to overcome low vaccination rates among HCP [52,84]. Recent studies indicate that mandatory vaccinations were supported by nearly 80% of hospital-based HCP and up to 90% of medical and dental university students in Europe [85,86,87]. Similarly, 67.3% of 250 HCP in three hospitals in the Democratic Republic of Congo believe that HBV vaccination should be mandatory [47]. The management of non-responders and infected HCP in the context of non-discrimination policies, should also be anticipated.

A primary three-dose vaccination series (0, 1, and 6 months) followed by serologic proof of adequate antibody response (anti-HBs titer of ≥10 IU/mL) at the time of first employment is the state of the art for long-term protection against hepatitis B in immunocompetent HCP [88]. The effectiveness of the three-dose vaccination series in HCP confirmed by an adequate antibody response has been proved in a recent meta-analysis [89]. HCP with underlying chronic conditions should be managed accordingly [90]. Overall, an analysis of costs associated with NSSIs notified from 2006 to 2016 in the Midwest region of Brazil, where 25,367 HCP are employed, showed that the direct costs of serologic confirmation following a primary vaccination series are significantly less than the costs of PEP for exposed HCP [91]. However, not all HCP seroconvert after a full vaccination series and chronic HBV infection has been occasionally reported in non-responsive HCP [92,93]. The age of primary hepatitis B vaccination influences the antibody response [77,94]. A study of 11,188 healthcare students in Italy from 2004 through 2020 found that approximately half of those who had received a primary vaccination series before the age of one year had a titer of anti-HBs <10 IU/mL, compared to 12.8% of students vaccinated after the age of one year [77]. Similarly, a study among medical students and HCP in India found a significant decline in antibody titers as the time after primary vaccination elapses (antibody titers were >10 IU/mL in 94.1% of HCP vaccinated ≤ 5 years, 79.7% in those after 6–10 years from primary vaccination, and 72.7% in those vaccinated >10 years) [95].

An antibody titer of <10 IU/mL does not necessarily imply loss of protection, since immunological memory may persist, as observed in boosted individuals [94]. For instance, a booster dose administered to 18-year-old healthcare students who were vaccinated against HBV in their first year of life was associated with a significant increase of the rate of students with antibody titers ≥ 10 IU/mL four years later (88.1% versus 41.3% in those vaccinated during infancy only, *p*-value < 0.001) [96]. In countries with high rates of childhood hepatitis B vaccination, estimating the HBV serostatus at the time of employment may be unnecessary. For instance, in an academic medical center in the United States (US), 51% (507 of 986) of HCP with a documented completion of the hepatitis B vaccination series as children had anti-HBs < 10 IU/mL [97]. Of them, 446 (88%) received a fourth hepatitis B vaccine shot, of whom only 11% (50 out 446 or 5% of all HCP) still had anti-HBs < 10 IU/mL [97]. Similarly, an anti-HBs antibody titer of <10 IU/mL was found at the time of first employment in approximately 90% of 734 individuals vaccinated during early childhood; nevertheless, their antibody response after a booster dose indicates that this is attributed to the natural decline in antibodies over time [98]. Therefore, retesting HBV vaccine responders is unnecessary after documentation of a protective antibody titer after the primary vaccination series [98]. A recent study reported an antibody response in 92.3% of HCP vaccinated with the highly immunogenic adjuvanted vaccine (Fendrix) and an excess raise in antibody titers after the first vaccine doses [99]. The latter vaccine can be considered for non-responsive HCP after two full vaccination series [99].

An accelerated hepatitis B vaccination course may be used to shorten the unprotected period in susceptible HCP at high risk for HBV infection. Data from the United Kingdom indicate that four months after a three-dose accelerated course of hepatitis B vaccination, 93% of the HCP developed antibody levels > 10 IU/mL, while after two courses (in case of non-response to the first vaccination course) almost 99% of them elicited an adequate antibody response [100]. Likewise, a decision-analytic model using real-world data from 23,004 US military recruits showed that after two doses (0, 1 month) with the newer adjuvant hepatitis B vaccine Heplisav-B, 92% of them elicited an antibody response within one month, compared with 24% if using the first-generation adjuvated hepatitis B vaccines [101]. The latter vaccination strategy significantly shorted the unprotected period by five months (between doses 2 and 3) and also saved 17.3% of costs [101]. The newer adjuvanted vaccine confers higher seroprotection within one month and may also result in higher vaccination rates, compared with the three-dose vaccination schedule [102]. Although US health economics models indicate cost-effectiveness for HCP over the three-dose vaccination scheme [102], cost-effectiveness studies should also be conducted in low-income countries.

Lastly, protocols for the storage and administration of vaccines should be in place. A study in Cameroon found that only two of three vaccinated individuals developed the level of 10 mIU/mL of anti-HBs 1–2 months after the completion of the three-dose vaccination schedule [103]. Gaps in the storage and administration of HBV vaccines were disclosed in all vaccination centers (e.g., the date vaccines were received, lot numbers, and name of vaccinators were not recorded; the temperature of the fridge was not monitored; contingency plans for vaccine storage in case of power or equipment failure were not issued) [103].

### 2.6. Infection Prevention Capacity

To reduce the prevalence of HBV infection among HCP, strategies to prevent, detect, and manage occupational exposure to HBV are needed. This is even more critical in the post-COVID-19 era, given the decreasing routine vaccination rates in children, particularly in several low-income countries that were already facing fragile vaccination programs [104]. Disruptions of infrastructure, supply chains, services, and interventions for HBV that maximized during the COVID-19 pandemic are expected to disproportionately affect the prevalence of chronic hepatitis B, particularly in low-income countries, providing a pool of transmission to future generations [105]. Accidental NSSIs are frequent among HCP and predisposing risk factors should be properly identified and addressed [106]. The incorrect notification of accidental NSSIs is also frequent [106]. Protective equipment, PEP, and guidelines for PEP should be available and communicated to HCP.

Lastly, medical waste generated in healthcare settings has increased considerably over the past decades [107]. Medical waste and particularly improperly handled and disposed needles and syringes pose a risk for the transmission of HBV [108]. The improper management of medical waste is frequent in developing countries, along with insufficient human resources, a lack of risk awareness, and gaps in training [108,109]. Written policies for medical waste management should be in place and communicated, and HCP should be educated accordingly [108].

Continuous training on infection prevention procedures for all HCP is imperative. Low-cost educational sessions to raise HBV knowledge and awareness among HCP were successfully implemented in Tanzania in recent years and may be used in similar low-resourced settings [110]. Likewise, training programs to promote occupational safety in the workplace and particularly to prevent NSSIs among HCP, of whom 40% had sustained NSSIs in the past, were successfully implemented in five Caribbean countries in the past decade [111]. These programs included lectures, workshops, policy reviews, and on-site evaluations of practices [111].

## 3. Conclusions

Despite the advances that have been made during the past three decades in vaccines, infection prevention, and PEP, hepatitis B continues to be a serious problem for HCP in many countries globally. New strategies to prevent and manage accidental exposure to blood and body fluids are needed in order to reduce the prevalence of HBV among HCP. Health authorities in several low-income countries that were already facing fragile vaccination programs should consider mandatory vaccination policies, particularly for HCP. Continuous training on infection prevention procedures for students in healthcare-related institutions and all HCP should be provided.

## Figures and Tables

**Table 1:** Characteristics of studies reporting data on HCP and hepatitis B, 2017–30 April 2023.

Study [Ref]	Country/Study Period	Setting/Data Collection	No and Characteristics of HCP	Findings
Nayyar et al. [12]	India, 2008–2014	pediatric tertiary care hospital/ blood testing, Questionnaire	739 HCP (21.1% physicians, 37.4% nurses, 12.8% technical staff, 15.6% nursing orderlies, 11.6% housekeeping staff, 1.2% others)	HBsAg (+): 0.6% fully vaccinated: 79.5%vaccinated housekeepers: 6.9%
Mercan Baspınar M [14]	Turkey, 2020	hospital/blood tests	1722 HCP (mean age: 34 years, 48.6% males, 55% doctors and nurses)	HBsAg (+): 0.7% AntiHBs > 10: 87.5% AntiHbs < 10: 12.5%
Tavoschi et al. [29]	EU/EEA countries, 2005–2017	38 studies, 4 on HCP (3 in Poland, 1 in Romania)		HBsAg (+) HCP: 06–1.2% in Poland, 2.2% in Romania
Hiva et al. [21]	Iran, 2017	public hospitals/ blood tests, questionnaire	776 HCP (mean age: 33.7 years, 75.72% females)	HBsAg (+):0.78% Anti-core (+): 4.64% Vaccinated: 100% NSSI: 42.4% past year
Al-Amad et al. [27]	United Arab Emirates, 2010–2017	university dental hospital/blood tests		88% of dental HCP were vaccinated
Ijoma et al. [19]	Nigeria, 2016	teaching hospital/ rapid tests, questionnaire	3123 HCP (mean age: 39.4 years)	HBsAg (+): 2.3%HCP good knowledge of the disease
Hebo et al. [3]	Ethiopia, 2015–2016	university hospital/ blood tests, questionnaire	240 HCP (mean age: 25 years, 50.4% males)	HBsAg (+): 2.5% 60% had history of exposure Most HCP good knowledge of disease
Yizengaw et al. [26]	Ethiopia, 2017	primary hospitals/ blood tests, questionnaire	338 HCP (120 medical waste handlers, mean age: 28.3 years, 54.9% males)	HBsAg (+): 2.6% in HCP, 2.5% in medical waste handlers
Kisangau et al. [9]	Kenya, 2017	hospitals, healthcare facilities/blood tests, questionnaire	312 HCP (median age: 31 years, 66% females)	HBsAg (+): 4% Anti HBs > 10: 47% Fully vaccinated: 48% Received ≥ 1 dose: 80%
Lieb et al. [16]	Liberia, 2017–2018	national medical center/blood tests	245 HCP (mean age: 37.9 years, 27.7% males)	HBsAg (+): 4.89%
Elzouki et al [18]	Libya, 2020	referral hospitals/ blood tests, questionnaire	182 HCP (mean age: 32.9 years, 78.6% females)	HBsAg (+): 4.9% Fully vaccinated: 52%NSSI: 54.9%
Maamor et al. [30]	Africa and Asia, 1997–2021	review of 25 studies, 19 from Africa and six from Asia	10,043 physicians, dentists, nurses, laboratory workers, technicians, nurse assistants, cleaning operators, and housekeeping staff	Seroprevalence: 5% (higher in Africa) In 11 studies association of seroprevalence and level of awareness
Mangkara et al. [20]	Vietnam, 2018	186 private dental clinics/blood test, questionnaire	206 dentists and 111 dental workers (age: 18–63 years)	HBsAg (+): 5% Immune due to vaccination: <15% Susceptible: 66.8% Low awareness, low knowledge about PEP
Yazie et al. [32]	Ethiopia, 2010–2019	6 articles on HCP	1747 HCP	prevalence: 5% (95% CI: 5–8%)
Mabunda et al. [2]	Mozambique, 2020	hospital/blood tests, questionnaire	315 HCP (39.7% nurses)	HBsAg/HBV DNA (+): 5.1% Immune due to vaccination: 60% Susceptible:30% HCP exposed to needles, blood, or other fluids had 2.13 times higher prevalence
Shao et al. [24]	Tanzania, 2015–2016	tertiary teaching hospital/blood tests, questionnaire	442 HCP (median age: 37 years, 60% females, 78.5% tertiary education, 56,1% non-surgical department	HBsAg (+): 5.7% 11.3% knew their HBV status physicians (24%), nurses (20%), lab personnel (16%) no satisfactory knowledge on HBV
Wijayadi et al. [4]	Indonesia, 2015–2016	blood tests, questionnaire	467 HCP (80.94% females)	HBsAg (+): 6.2% Anti-core (+):19.2% Anti HBs (+):26.1% No markers: 66.17% NSSI was the highest risk factor for infection
Atlaw et al. [31]	Africa, 1989–2020	44 studies	17,510 HCP	Prevalence: 6.81% (Western Africa: 11.67%, Northern Africa: 3.5%)
Massaquoi et al. [25]	Sierra Leone, 2017	tertiary teaching hospital, military general hospital/blood tests, questionnaire	447 HCP (90.6% nurses, 72.3% females)	HBsAg (+): 8.7% No significant difference by sex, age, work site or profession
Bilounga Ndongo C et al. [10]	Cameroon, 2016	16 hospitals/blood tests, questionnaire	1836 HCP (mean age: 34 years, 65.3% females)	HBsAg (+): 8.7% 11.4% at least 3 doses of HBV vaccine Patients’ transporters highest prevalence (14.3%), physicians the lowest (3.2%)
Nguyen et al. [15]	Vietnam, 2021	3 hospitals/blood tests, questionnaire	203 HCP (39 physicians, 140 nurses/midwives, 24 technicians/nurse assistants; mean age: 34.49 years	HBsAg (+): 9.85% Anti-core (+): 27.14% AntiHBs > 10: 74.87% Only 50% of infected HCP used gloves
Qin et al. [11]	Sierra Leone, 2017	34 military hospitals/blood tests, questionnaire	211 HCP (median age: 39 years, 51.2% males)	HBsAg (+): 10% Susceptible:81.5% Vaccinated:4.3% Immune due to past infection: 4.3%
Akazong et al. [17]	Cameroon, 2017	22 healthcare facilities/blood tests, questionnaire	395 HCP (68.4% females)	HBsAg (+): 10.6% Vaccinated: 9.1% Susceptible: 43.5%
El Bara et al. [8]	Mauritania, 2014–2016	13 districts/ blood tests	3857 HCP (medical and paramedical staff)	HBsAg (+): 10.8% Anti-core (+): 64.6%Anti-HBs: 32.3%
Machange et al. [13]	Tanzania, 2014	medical center/blood tests, questionnaire	76 HCP (range: 20–56 years, 52.6% males)	HBsAg (+): 11.8%Vaccinated: 10.5%Low knowledge of risks for HBV infection
Ganczak et al. [23]	Poland, 2016–2018	10 hospitals/blood tests, questionnaire	306 HCP (mean age: 47.7 years, 69.9% nurses, 88.6% females)	Anti-core (+): 12.1% 10.5% of vaccinated were anti-core (+) Vaccinated: 94.2%, 76.1% no post vaccine serology
Domínguez et al. [28]	Spain, 2008–2010	primary healthcare, hospital/blood tests, questionnaire	644 HCP (median age: 42 years, 23.4% males, 46.4% primary care, 53.6% hospital HCP)	Anti-core (+): 4.1% Anti HBs (+): 64.4%prevalence of anti-core increased with age

CI: confidence interval; EU/EEA: European Union/European Economic Area; HCP: healthcare personnel; No: number; NSSI: needle stick and sharps injury; PEP: post-exposure prophylaxis; Ref: reference.

## Data Availability

Not applicable.
